# Association of Perioperative Shock Indices with In-Hospital Mortality and Postoperative Morbidity After Coronary Artery Bypass Grafting: A Retrospective Cohort Study

**DOI:** 10.3390/jcm15218629

**Published:** 2026-11-05

**Authors:** Selen Topalel, Mehmet Özkılıç

**Affiliations:** Department of Anesthesiology and Reanimation, Gazi Yaşargil Training and Research Hospital, University of Health Sciences, Diyarbakır 21070, Türkiye

**Keywords:** coronary artery bypass, hospital mortality, modified shock index, postoperative complications, risk assessment, shock index

## Abstract

**Background/Objectives:** Shock index (SI) and modified shock index (MSI) are simple hemodynamic indices derived from routinely available vital signs, but their perioperative prognostic relevance in coronary artery bypass grafting (CABG) remains incompletely characterized. This study evaluated perioperative changes in SI, MSI, and their age-adjusted derivatives and explored their associations with in-hospital mortality and postoperative outcomes after elective isolated CABG. **Methods:** This retrospective single-center study included 245 adults who underwent elective isolated CABG between 1 January 2023, and 1 May 2025. SI, MSI, Age-SI, and Age-MSI were calculated at seven perioperative time points. The primary outcome was in-hospital mortality; prespecified secondary outcomes included AKI, stroke, ICU length of stay, and mechanical ventilation duration, while additional postoperative morbidity outcomes were explored. Discrimination was assessed using receiver operating characteristic analysis. Exploratory models evaluated post-cardiopulmonary bypass (post-CPB) SI and MSI in combination with EuroSCORE II, with Firth penalized regression and bootstrap internal validation used as sensitivity analyses because of the limited number of deaths. **Results:** In-hospital mortality occurred in 12/245 patients (4.9%). Post-CPB measurements were available in 243 patients, including 10 non-survivors. Among the evaluated time points, the strongest observed mortality discrimination occurred post-CPB: SI had an AUC of 0.798 (95% CI 0.621–0.974) and MSI an AUC of 0.793 (95% CI 0.606–0.980). After adjustment for EuroSCORE II, both post-CPB SI (OR 1.30 per 0.1-unit increase, 95% CI 1.09–1.56; *p* = 0.004) and MSI (OR 1.23 per 0.1-unit increase, 95% CI 1.07–1.41; *p* = 0.003) remained associated with mortality. Addition of either index increased the apparent AUC from 0.798 to 0.830 and improved model fit; however, the absolute increase in discrimination was modest, and bootstrap confidence intervals for ΔAUC included zero. Firth penalized regression yielded similar effect estimates, and optimism-corrected AUCs were 0.824 for EuroSCORE II + SI and 0.829 for EuroSCORE II + MSI. Age-adjusted indices showed exploratory associations with several postoperative morbidity outcomes. **Conclusions:** Post-CPB SI and MSI were associated with in-hospital mortality among patients who reached the post-CPB assessment point and remained associated after adjustment for EuroSCORE II. Although their addition improved model fit, incremental improvement in discrimination was modest and uncertain. These readily available indices may serve as supportive markers of perioperative hemodynamic instability rather than standalone prognostic tools. Given the limited number of mortality events, these findings should be considered exploratory and require external validation.

## 1. Introduction

Accurate perioperative risk assessment remains an important component of care in patients undergoing coronary artery bypass grafting (CABG). EuroSCORE II is an established risk model for estimating operative mortality after cardiac surgery [1]. However, as a preoperative risk model, it does not capture the dynamic hemodynamic changes that occur during cardiopulmonary bypass (CPB) and the early postoperative period. Simple measures derived from routinely monitored hemodynamic variables may therefore provide complementary information by reflecting the patient’s physiological status at different stages of the perioperative course.

The shock index (SI), calculated as the ratio of heart rate to systolic blood pressure, is a simple measure of hemodynamic status. The modified shock index (MSI) uses mean arterial pressure instead of systolic blood pressure, while age-adjusted derivatives incorporate age into SI or MSI. These indices can be calculated from routinely available vital signs without additional laboratory testing or specialized monitoring, making them suitable for repeated bedside assessment [2,3].

The prognostic value of SI and its derivatives has been investigated in several cardiovascular populations. Associations with mortality and other adverse outcomes have been reported in patients with acute myocardial infarction and ST-segment elevation myocardial infarction (STEMI), and both modified and age-adjusted indices have been evaluated for cardiovascular risk stratification [4]. Age-SI and Age-MSI have also been compared with established risk models such as the Global Registry of Acute Coronary Events (GRACE) score in patients undergoing percutaneous coronary intervention [5]. More recently, SI-based indices have been evaluated in patients with acute and chronic heart failure, extending their application beyond acute coronary syndromes [6,7].

In contrast, evidence regarding the use of shock indices in cardiac surgical patients remains limited. In a recent study of adults undergoing cardiac surgery with cardiopulmonary bypass (CPB), SI measured at ICU admission was independently associated with subsequent hemodynamic failure [8]. Earlier studies in acute myocardial infarction and STEMI populations also reported prognostic associations for age-adjusted shock indices [9,10]. However, the perioperative prognostic value of SI and its modified and age-adjusted derivatives has not been well characterized in patients undergoing isolated CABG. Moreover, it remains unclear whether their prognostic value varies across different perioperative time points or whether they provide information complementary to established preoperative risk assessment.

The present study therefore evaluated SI, MSI, Age-SI, and Age-MSI at multiple perioperative time points in patients undergoing elective isolated CABG. The primary aim was to investigate their association with in-hospital mortality and to identify the perioperative time point with the greatest prognostic discrimination. Secondary analyses examined their associations with postoperative morbidity and assessed whether the most informative indices provided prognostic information beyond EuroSCORE II. We hypothesized that perioperative shock indices would be associated with in-hospital mortality and that their prognostic performance might vary across measurement time points.

## 2. Methods

### 2.1. Study Design and Setting

This single-center, retrospective observational cohort study evaluated the association of perioperative shock indices with in-hospital mortality and postoperative morbidity in adult patients undergoing isolated coronary artery bypass grafting (CABG). The study included patients treated between 1 January 2023, and 1 May 2025.

The study protocol and statistical analysis plan were preregistered on the Open Science Framework (OSF; DOI: 10.17605/OSF.IO/5MDXU) before any study-specific data extraction, compilation, or analysis and before the investigators examined the relationships between the hemodynamic indices and clinical outcomes.

The study was approved by the University of Health Sciences Gazi Yaşargil Training and Research Hospital Clinical Research Ethics Committee (approval no. 483, 23 May 2025) and was conducted in accordance with the principles of the Declaration of Helsinki.

### 2.2. Study Population

We screened the medical records of 427 adults who underwent cardiac surgery during the study period. Patients aged ≥18 years who underwent elective isolated CABG were included in the analysis. Patients undergoing other cardiac surgical procedures, CABG combined with another cardiac procedure, or emergency CABG were excluded.

Among the 427 patients screened, 162 underwent either non-CABG cardiac surgery or CABG with a concomitant cardiac procedure, and 20 underwent emergency isolated CABG. The final cohort therefore comprised 245 patients who underwent elective isolated CABG. All eligible patients treated during the study period were included. Because the study used all available eligible cases, no formal a priori sample-size calculation was performed.

### 2.3. Data Sources and Collection

Eligible patients were identified from the institutional surgical registry. Data were retrospectively obtained from the Hospital Information System, electronic medical records, anesthesia records, and cardiovascular surgery ICU charts and entered into an electronic case report form. Baseline variables included age, sex, body mass index (BMI), body surface area (BSA), New York Heart Association (NYHA) functional class, left ventricular ejection fraction (LVEF), EuroSCORE II, diabetes mellitus, hypertension, preoperative renal failure, previous cerebrovascular events, pulmonary arterial hypertension, preoperative atrial fibrillation, smoking status, and chronic obstructive pulmonary disease (COPD). Preoperative laboratory data included hemoglobin, platelet count, international normalized ratio (INR), urea, and serum creatinine.

Intraoperative data included aortic cross-clamp and CPB durations, anesthesia and surgical durations, urine output, peak lactate concentration, and the use of vasoactive or inotropic agents. Vasoactive/inotropic exposure was available as treatment-use indicators, including intraoperative norepinephrine and dopamine use; detailed dose, cumulative exposure, infusion rate, titration, and time-resolved administration relative to the post-CPB hemodynamic measurements were not consistently available in the retrospective records. Heart rate (HR), systolic blood pressure (SBP), and mean arterial pressure (MAP) were obtained from anesthesia and cardiovascular surgery intensive care unit (ICU) monitoring records for calculation of the shock indices.

Hemodynamic measurements were assessed at seven perioperative time points: before induction of anesthesia (pre-induction), before initiation of CPB (pre-CPB), after separation from CPB and protamine administration (post-CPB), at ICU admission (0 h), and at 6, 12, and 24 h after surgery. Pre-induction, post-CPB, 0 h, 6 h, and 24 h were prespecified in the preregistered protocol. Pre-CPB and 12 h measurements were additionally available in the records and were analyzed as exploratory time points.

Each patient was assigned a study identifier, and direct personal identifiers were excluded from the analytical dataset.

### 2.4. Cardiopulmonary Bypass Management

All procedures were performed using conventional extracorporeal circulation, as minimally invasive extracorporeal circulation (MiECC) was not available at our institution during the study period. Cardiopulmonary bypass was conducted using a roller pump and a hollow-fiber membrane oxygenator. Pump flow was targeted at approximately 2.4 L/min/m^2^ under normothermic conditions and approximately 1.8–2.0 L/min/m^2^ during moderate hypothermia at 32 °C. Mean arterial pressure during CPB was generally maintained between 45 and 75 mmHg. Mild-to-moderate hypothermia (32–34 °C) was routinely employed. These parameters reflected the institutional perfusion strategy applied during the study period.

### 2.5. Calculation of Shock Indices

Four hemodynamic indices were calculated at each perioperative time point using simultaneously recorded HR, SBP, and MAP values:

Shock Index (SI) = HR/SBP

Modified Shock Index (MSI) = HR/MAP

Age Shock Index (Age-SI) = Age × SI

Age-Modified Shock Index (Age-MSI) = Age × MSI

The calculated indices were not available to the treating clinical teams and did not influence perioperative management.

### 2.6. Study Outcomes

The prespecified primary outcome was in-hospital mortality. Associations of perioperative SI, MSI, Age-SI, and Age-MSI with in-hospital mortality were evaluated at the available measurement time points. Prespecified secondary outcomes included ICU length of stay, duration of mechanical ventilation, postoperative acute kidney injury (AKI), and postoperative stroke. Additional exploratory postoperative outcomes included new-onset postoperative atrial fibrillation (AF), surgical re-exploration, reintubation, and overall postoperative morbidity (OPM). OPM was defined as the occurrence of at least one postoperative complication during the index hospitalization, including hypertension or hypotension, AKI, stroke, AF, surgical re-exploration, respiratory complications or respiratory failure (including pleural effusion and pneumothorax), bleeding, surgical-site infection, or reintubation. Because postoperative blood-pressure abnormalities overlap conceptually with shock indices, a sensitivity analysis used a modified OPM definition that excluded postoperative hypertension and hypotension from the composite. Frequencies of the individual components of OPM were also evaluated separately.

Postoperative AKI was classified using a creatinine-based, KDIGO-derived staging approach based on the fold increase in serum creatinine relative to the preoperative baseline value. Stage 1 was defined as a ≥1.5-fold increase in serum creatinine, Stage 2 as a ≥2.0-fold increase, and Stage 3 as a ≥3.0-fold increase or the requirement for renal replacement therapy. Patients not meeting these criteria were classified as Stage 0. The additional KDIGO criterion of an absolute increase in serum creatinine of ≥0.3 mg/dL within 48 h was not incorporated into the prespecified study classification. For the principal AKI analyses, patients were categorized as no AKI (Stage 0) versus AKI (Stages 1–3); analyses across individual stages were exploratory.

The post-CPB shock-index assessment was obtained after separation from CPB and protamine administration and before transfer to the cardiovascular surgical ICU. Postoperative AF, surgical re-exploration, and other postoperative complications were identified during the subsequent index hospitalization. Because the exact timing of all individual postoperative events relative to the post-CPB measurement was not available with sufficient granularity for formal time-to-event analyses, post-CPB values were used as the principal early measurements for analyses of these outcomes. Associations with these outcomes were interpreted as exploratory associations rather than causal or time-to-event relationships, and analyses involving later postoperative measurements were not interpreted as predictive when the temporal sequence between the index measurement and outcome could not be established.

### 2.7. Statistical Analysis

Continuous variables were assessed for distributional characteristics using the Kolmogorov–Smirnov test and visual inspection where appropriate. As continuous variables did not demonstrate a normal distribution, they are presented as median [interquartile range] and were compared using the Mann–Whitney U test. Categorical variables are presented as number (percentage) and were compared using the chi-square test or Fisher’s exact test, as appropriate.

Perioperative SI, MSI, Age-SI, and Age-MSI values were compared between survivors and non-survivors at each of the seven measurement time points. Analyses at each time point were based on available observations. Measurements that were structurally unavailable because a patient had not reached the defined assessment point were not imputed. Specifically, the post-CPB measurement required successful separation from CPB and protamine administration; therefore, patients who could not be successfully separated from CPB had no post-CPB value. Similarly, measurements scheduled after a patient’s death were unavailable by definition. Because these patterns of missingness were related to the clinical course and potentially to the outcome, analyses of post-CPB and later postoperative measurements were interpreted with consideration of informative missingness.

Receiver operating characteristic (ROC) curve analysis was used to assess discrimination for in-hospital mortality and selected secondary outcomes. Areas under the ROC curve (AUCs) are reported with 95% confidence intervals (CIs). For descriptive purposes, exploratory cut-off values were identified using the Youden index, and sensitivity, specificity, positive predictive value, and negative predictive value were calculated. These internally derived cut-offs were not considered validated clinical decision thresholds. The discriminatory performance of post-CPB shock indices was additionally compared with EuroSCORE II using a common complete-case–cohort. Pairwise AUC comparisons were performed using the DeLong method. To explore whether post-CPB shock indices provided information beyond EuroSCORE II, separate logistic regression models were constructed for EuroSCORE II alone and for EuroSCORE II combined with either post-CPB SI or MSI. Each incremental model therefore included two predictor terms, EuroSCORE II and one shock index, in addition to the intercept. Model discrimination was assessed by AUC, and the contribution of SI or MSI was evaluated using likelihood-ratio tests.

Given the small number of deaths, these analyses were considered exploratory and the models were deliberately kept parsimonious. As a sensitivity analysis for potential small-sample bias, the EuroSCORE II + SI and EuroSCORE II + MSI models were additionally fitted using Firth penalized logistic regression. Internal validation was performed using 1000 stratified bootstrap resamples. Bootstrap resampling was used to estimate model optimism and optimism-corrected AUC and Brier score. Calibration was additionally assessed using bootstrap-validated calibration slopes. Uncertainty in the change in AUC after addition of SI or MSI was assessed using bootstrap confidence intervals.

Multivariable logistic regression was performed selectively when the number of outcome events allowed a parsimonious model. Adjusted odds ratios (aORs) with 95% CIs were reported. SI and MSI were modeled per 0.1-unit increase and Age-SI and Age-MSI per 10-unit increase. Adjusted secondary-outcome models included EuroSCORE II and the indicated post-CPB shock index only. Multivariable analysis was not performed for reintubation because of the small number of events. The modified OPM sensitivity analysis used the same approach as the original OPM analysis.

Associations of post-CPB shock indices with ICU length of stay and mechanical ventilation duration were assessed using Spearman rank correlation. AKI was primarily analyzed as a binary outcome (Stage 0 vs. Stages 1–3); differences across individual AKI stages were additionally evaluated using the Kruskal–Wallis test, and associations with ordinal AKI severity were assessed using Spearman rank correlation.

All tests were two-sided, and *p* < 0.05 was considered statistically significant. Analyses of additional postoperative outcomes and non-preregistered time points were exploratory. No formal adjustment for multiple comparisons was applied to these exploratory analyses, and their findings were considered hypothesis-generating.

Analyses were performed using IBM SPSS Statistics version 24.0 (IBM Corp., Armonk, NY, USA) and Python version 3.13.5 (Python Software Foundation, Beaverton, OR, USA), with SciPy version 1.17.0, statsmodels version 0.14.6, and scikit-learn version 1.8.0.

## 3. Results

### 3.1. Study Population and Baseline Characteristics

**Figure 1:**
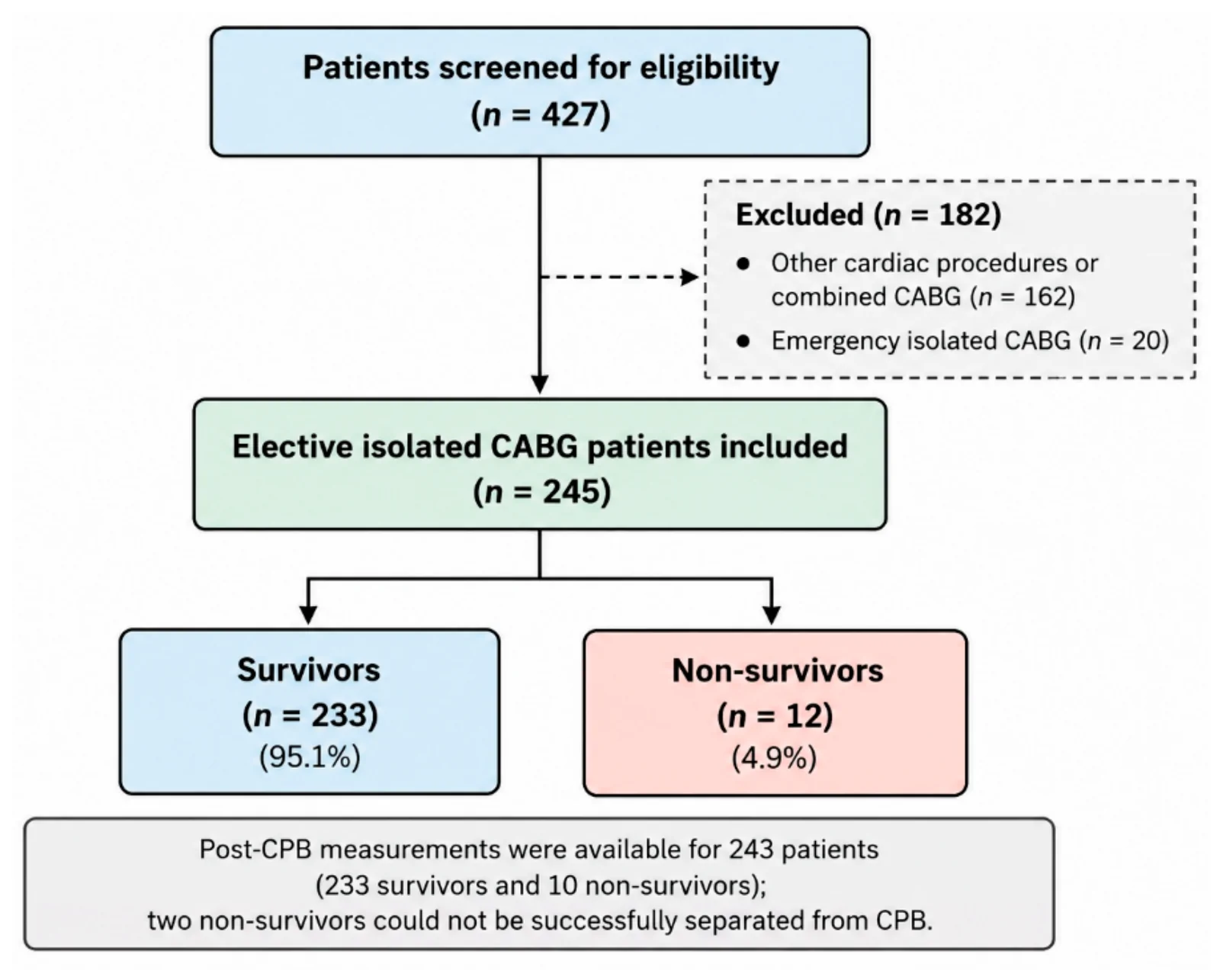
Flow diagram of patient selection.

The final study cohort comprised 245 patients undergoing elective isolated CABG (Figure 1). In-hospital mortality occurred in 12 patients (4.9%), while 233 patients (95.1%) survived to hospital discharge.

Baseline and intraoperative characteristics according to in-hospital mortality are presented in Table 1. Compared with survivors, non-survivors had a more advanced NYHA functional class (*p* < 0.001), lower LVEF (50 [41.3–55]% vs. 55 [50–60]%; *p* = 0.023), and higher EuroSCORE II (2.93 [1.61–6.56]% vs. 1.08 [0.80–1.59]%; *p* < 0.001). Preoperative renal failure was more frequent among non-survivors (25.0% vs. 3.0%; *p* = 0.009), who also had higher preoperative urea (53 [40–76] vs. 38 [31–45] mg/dL; *p* = 0.007) and creatinine levels (1.03 [0.84–2.30] vs. 0.82 [0.70–1.00] mg/dL; *p* = 0.011). BSA was lower in non-survivors (1.81 [1.70–1.84] vs. 1.87 [1.77–1.97] m^2^; *p* = 0.043). Among intraoperative variables, norepinephrine use was more frequent in non-survivors (100% vs. 60.1%; *p* = 0.004). Other baseline and intraoperative characteristics did not differ significantly between groups.

### 3.2. Perioperative Trajectories of Shock Indices and In-Hospital Mortality

**Figure 2:**
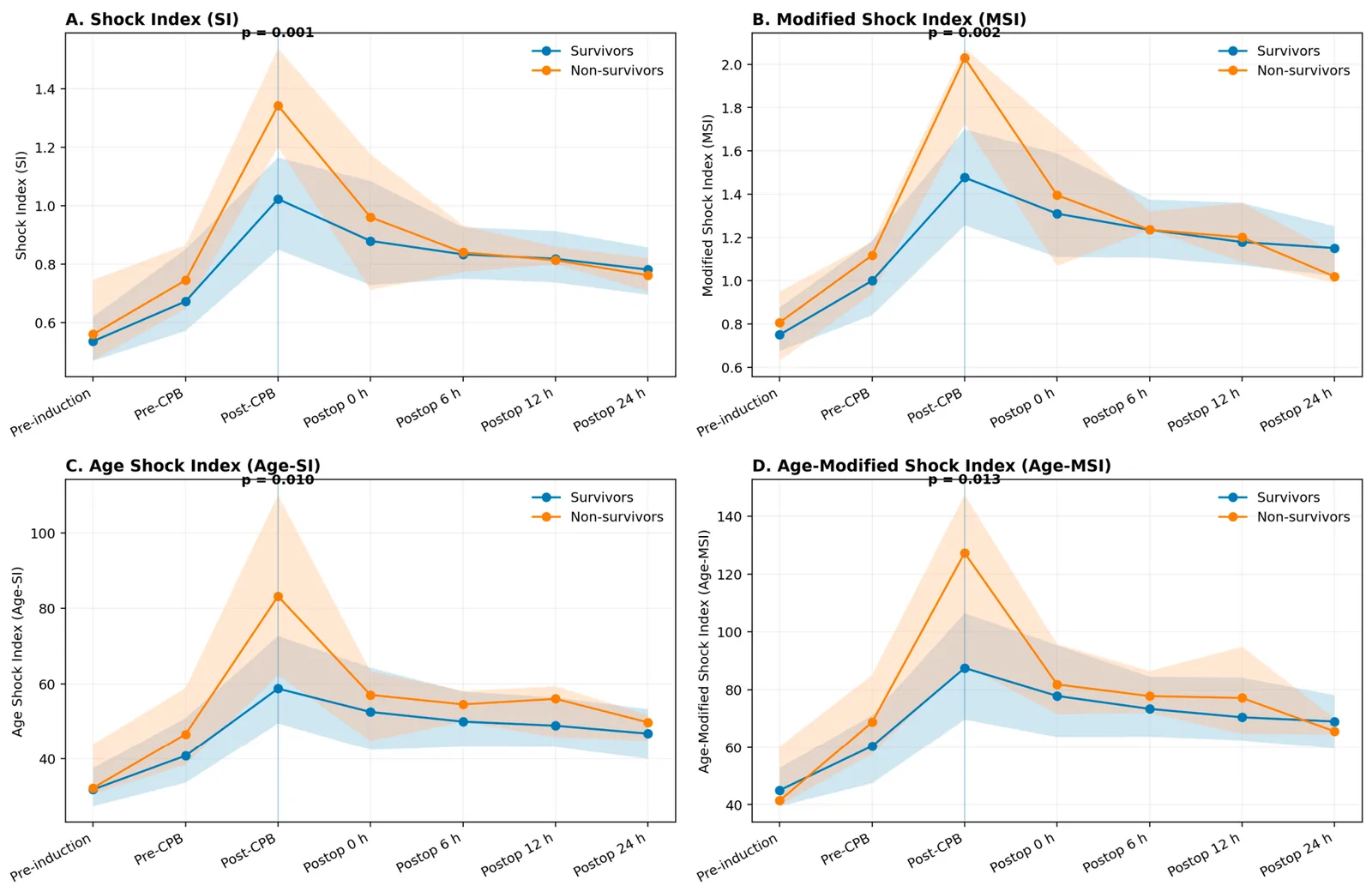
Perioperative trajectories of shock indices according to in-hospital mortality. Median values and interquartile ranges of (**A**) shock index (SI), (**B**) modified shock index (MSI), (**C**) age shock index (Age-SI), and (**D**) age-modified shock index (Age-MSI) are shown according to the available observations at each perioperative time point. *p* values shown in the panels refer to between-group comparisons at the post-CPB assessment. The number of available observations varied according to index and time point and is reported in Supplementary Table S1. Two non-survivors did not have post-CPB measurements because they could not be successfully separated from CPB. Subsequent deaths further reduced the number of available observations at later postoperative time points; therefore, later trajectories should be interpreted descriptively.

Post-CPB measurements were available for 243 patients (233 survivors and 10 non-survivors); two non-survivors could not be successfully separated from CPB and therefore had no post-CPB measurement. The number of available measurements varied by index and time point. Subsequent deaths further reduced the number of available observations among non-survivors; therefore, later postoperative trajectories were interpreted descriptively rather than as evidence that later measurements were intrinsically less prognostic. The perioperative trajectories of the four shock indices according to in-hospital mortality are presented in Figure 2.

### 3.3. Discrimination of Post-CPB Shock Indices for In-Hospital Mortality

**Figure 3:**
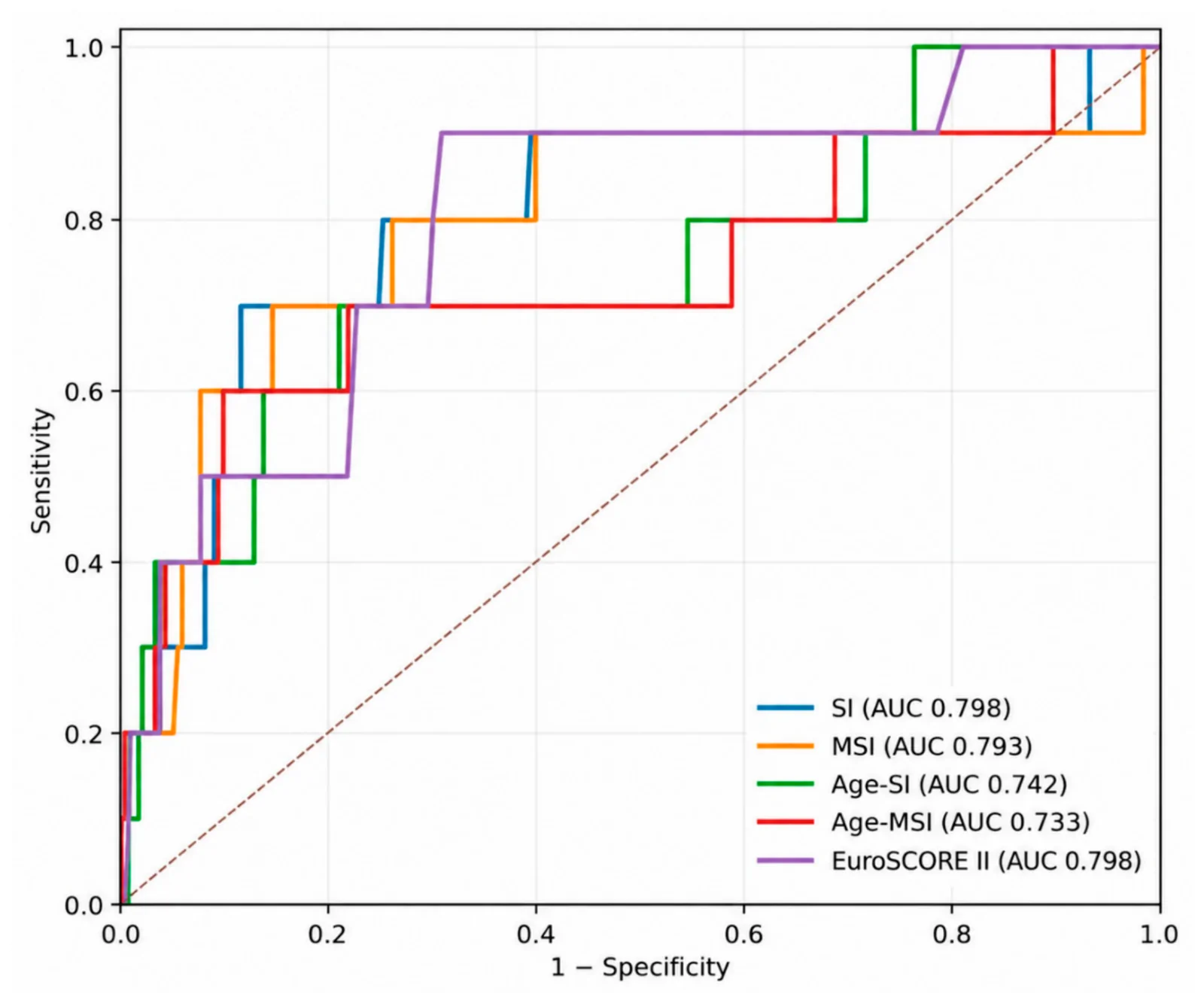
Receiver operating characteristic curves of post-CPB shock indices and EuroSCORE II for in-hospital mortality in the common complete-case–cohort. All curves were generated using the same 243 patients with available post-CPB measurements, including 10 deaths. In this common cohort, the AUCs were 0.798 for SI, 0.793 for MSI, 0.742 for Age-SI, 0.733 for Age-MSI, and 0.798 for EuroSCORE II. The EuroSCORE II AUC reported here therefore differs from the whole-cohort estimate of 0.830 reported in Table 2. The red dashed diagonal line represents the reference line of no discrimination (AUC = 0.50). AUC: area under the receiver operating characteristic curve; CPB: cardiopulmonary bypass; SI: shock index; MSI: modified shock index; Age-SI: age shock index; Age-MSI: age-modified shock index; EuroSCORE II: European System for Cardiac Operative Risk Evaluation II.

The discriminatory performance of the post-CPB shock indices for in-hospital mortality is presented in Table 2 and Figure 3. Post-CPB SI demonstrated an AUC of 0.798 (95% CI 0.621–0.974). The exploratory cut-off of ≥1.31 yielded 70.0% sensitivity, 88.4% specificity, a PPV of 20.6%, and an NPV of 98.6%. Post-CPB MSI showed similar discrimination, with an AUC of 0.793 (95% CI 0.606–0.980); the exploratory cut-off of ≥1.87 yielded 70.0% sensitivity, 85.4% specificity, a PPV of 17.1%, and an NPV of 98.5%.

The age-adjusted indices showed lower discrimination. Post-CPB Age-SI had an AUC of 0.742 (95% CI 0.554–0.929), with an exploratory cut-off of ≥76.87, whereas Age-MSI had an AUC of 0.733 (95% CI 0.527–0.939), with a cut-off of ≥126.69. Their corresponding NPVs were 98.4% and 98.1%, respectively. Post-CPB analyses included 243 patients (10 deaths); two non-survivors could not be successfully separated from CPB and therefore had no post-CPB measurement.

In the entire cohort (n = 245; 12 deaths), EuroSCORE II yielded an AUC of 0.830 (95% CI 0.698–0.963). An exploratory cut-off of ≥1.47 yielded 91.7% sensitivity, 69.1% specificity, a PPV of 13.3%, and an NPV of 99.4%.

### 3.4. Incremental Performance Beyond EuroSCORE II

**Figure 4:**
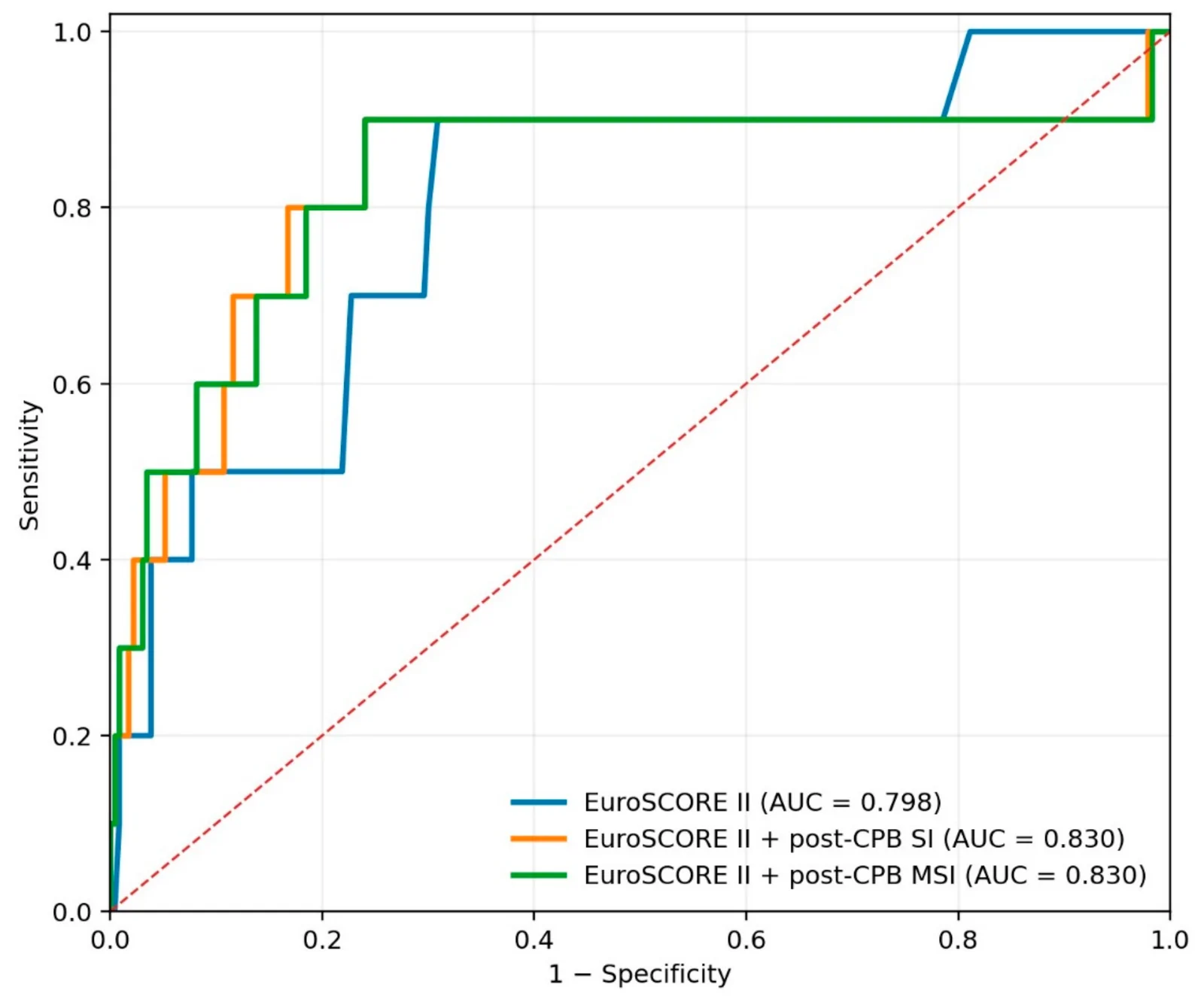
Incremental performance of post-CPB SI and MSI when added to EuroSCORE II for in-hospital mortality. Receiver operating characteristic curves compare EuroSCORE II alone with models including EuroSCORE II plus post-CPB SI or MSI in the common complete-case–cohort (n = 243; 10 deaths). The apparent AUC increased from 0.798 for EuroSCORE II alone to 0.830 after addition of either SI or MSI. Addition of SI or MSI improved model fit in likelihood-ratio testing; however, the 95% bootstrap confidence intervals for ΔAUC included zero (SI: −0.035 to 0.115; MSI: −0.032 to 0.118), indicating uncertainty regarding the magnitude of improvement in discrimination. The red dashed diagonal line represents the reference line of no discrimination (AUC = 0.50). AUC: area under the receiver operating characteristic curve; CPB: cardiopulmonary bypass; SI: shock index; MSI: modified shock index; EuroSCORE II: European System for Cardiac Operative Risk Evaluation II.

For direct comparison with EuroSCORE II, analyses were restricted to the common complete-case–cohort of 243 patients with 10 deaths. In this cohort, EuroSCORE II alone yielded an apparent AUC of 0.798. Addition of either post-CPB SI or MSI increased the apparent AUC to 0.830 (ΔAUC approximately 0.031 for both models) (Figure 4).

Post-CPB SI remained associated with in-hospital mortality after adjustment for EuroSCORE II (OR 1.30 per 0.1-unit increase, 95% CI 1.09–1.56; *p* = 0.004). Post-CPB MSI was similarly associated with mortality (OR 1.23 per 0.1-unit increase, 95% CI 1.07–1.41; *p* = 0.003). Likelihood-ratio testing showed improved model fit after addition of post-CPB SI (LR χ^2^ = 7.61; *p* = 0.0058) or MSI (LR χ^2^ = 8.57; *p* = 0.0034). Firth penalized logistic regression yielded similar estimates for SI (OR 1.29 per 0.1-unit increase, 95% CI 1.08–1.53; penalized LR *p* = 0.0056) and MSI (OR 1.22 per 0.1-unit increase, 95% CI 1.07–1.40; *p* = 0.0037).

In 1000 stratified bootstrap resamples, optimism-corrected AUCs were 0.797 for EuroSCORE II alone, 0.824 for EuroSCORE II + SI, and 0.829 for EuroSCORE II + MSI. Corresponding optimism-corrected Brier scores were 0.0380, 0.0361, and 0.0350, respectively. Bootstrap-validated calibration slopes for the two incremental models were approximately 0.95. Nevertheless, the 95% bootstrap intervals for ΔAUC included zero for both EuroSCORE II + SI (−0.035 to 0.115) and EuroSCORE II + MSI (−0.032 to 0.118). Thus, the indices improved model fit, but the magnitude of incremental improvement in discrimination remained modest and uncertain.

### 3.5. Postoperative Outcomes

Associations between post-CPB shock indices and secondary postoperative outcomes are summarized in Table 3. Postoperative AKI occurred in 28 of 244 evaluable patients (11.5%). Post-CPB Age-SI was higher in patients who developed AKI than in those who did not (75.32 vs. 58.45; *p* = 0.023), although discrimination was modest (AUC = 0.635). Exploratory analyses were also performed across individual KDIGO-derived AKI stages.

Postoperative AF occurred in 36 patients (14.7%). Age-SI and Age-MSI were associated with AF in univariable analyses (*p* = 0.037 and *p* = 0.028, respectively), but neither association remained statistically significant after adjustment (Age-SI: aOR 1.15 per 10-unit increase, *p* = 0.089; Age-MSI: aOR 1.11 per 10-unit increase, *p* = 0.070).

Surgical re-exploration occurred in 25 patients (10.2%). Age-SI and Age-MSI were associated with re-exploration in univariable analyses (both *p* < 0.001; AUC = 0.710 and 0.720, respectively), and both remained significant after adjustment (Age-SI: aOR 1.29 per 10-unit increase, 95% CI 1.08–1.53; *p* = 0.005; Age-MSI: aOR 1.22, 95% CI 1.07–1.39; *p* = 0.002).

Overall postoperative morbidity occurred in 155 patients (63.3%). Age-SI and Age-MSI were associated with OPM in univariable analyses (both *p* = 0.005; AUC = 0.607 for both) and remained associated after adjustment (Age-SI: aOR 1.23 per 10-unit increase, 95% CI 1.05–1.43; *p* = 0.008; Age-MSI: aOR 1.17, 95% CI 1.05–1.30; *p* = 0.005). In the sensitivity analysis excluding postoperative hypertension and hypotension from the composite, modified OPM occurred in 120/245 patients (49.0%; 119/243 among patients with available post-CPB measurements). Age-SI remained higher in patients with modified OPM (64.44 [51.99–80.55] vs. 55.70 [48.35–67.23]; *p* < 0.001; AUC = 0.627) and was associated with modified OPM after adjustment for EuroSCORE II (aOR 1.25 per 10-unit increase, 95% CI 1.08–1.44; *p* = 0.002). Corresponding results for Age-MSI were 93.53 [74.71–116.43] vs. 82.31 [68.54–96.59] (*p* < 0.001; AUC = 0.625) and aOR 1.17 per 10-unit increase (95% CI 1.06–1.29; *p* = 0.002).

Median ICU length of stay was 2 [2,3] days, with no significant correlation with any post-CPB shock index. Median duration of mechanical ventilation was 5.92 [4.50–7.00] h. SI and MSI were not significantly correlated with ventilation duration, whereas Age-SI and Age-MSI showed weak positive correlations (ρ = 0.146 for both; *p* = 0.024). Postoperative stroke occurred in 2 of 244 evaluable patients (0.8%); inferential analysis was not performed because only one patient with postoperative stroke had an available post-CPB measurement.

## 4. Discussion

In this retrospective cohort of patients undergoing elective isolated CABG, perioperative shock indices were associated with in-hospital mortality, with the strongest observed between-group differences occurring after separation from cardiopulmonary bypass. At this time point, SI and MSI showed numerically greater discrimination for mortality than their age-adjusted derivatives and remained associated with mortality after adjustment for EuroSCORE II, although their incremental prognostic contribution was modest. Age-adjusted indices also showed exploratory associations with selected postoperative complications, particularly surgical re-exploration and overall postoperative morbidity. Overall, these findings suggest that shock indices may serve as readily available markers of perioperative hemodynamic instability rather than as standalone prognostic tools.

The timing of shock-index measurement appears particularly relevant in cardiac surgery. In our cohort, the strongest observed differences between survivors and non-survivors occurred after separation from CPB. Importantly, two patients who died could not be successfully separated from CPB and therefore had no post-CPB measurement, while subsequent deaths produced outcome-dependent attrition at later time points. Consequently, the post-CPB estimates characterize patients who reached this assessment point, and weaker associations at later measurements should not be interpreted as evidence that later time points are intrinsically less prognostic. Garrido Callén et al. reported that a higher SI at ICU admission was independently associated with subsequent hemodynamic failure in patients undergoing cardiac surgery with CPB [8]. Although the measurement time points and outcomes differed, both studies support the potential relevance of hemodynamic indices around the transition from surgery to intensive care. At the same time, post-CPB heart rate and arterial pressure are influenced by ventricular recovery, volume status, anesthetic management, and vasoactive or inotropic support. In our cohort, intraoperative norepinephrine use was more frequent among non-survivors, but detailed dose and time-resolved exposure were unavailable. Shock indices at this stage should therefore be interpreted as integrated hemodynamic states under active perioperative treatment rather than direct measures of intrinsic myocardial function.

An important question is whether perioperative shock indices provide prognostic information beyond established cardiac surgical risk models. EuroSCORE II was developed to estimate operative mortality using preoperative patient characteristics and clinical risk factors [1]. In our cohort, both post-CPB SI and MSI remained associated with in-hospital mortality after adjustment for EuroSCORE II, and these associations were robust to penalized regression and internal validation. However, the improvement in discrimination after adding either index was modest and uncertain. As emphasized by Pencina et al., statistical association does not necessarily translate into clinically meaningful improvement in risk prediction [11]. Recent CABG research has likewise examined whether readily available markers can complement established surgical risk models. Sayarer et al. reported that the preoperative Naples prognostic score, reflecting inflammatory and nutritional status, was associated with mortality and improved predictive performance when incorporated into EuroSCORE II [12]. Although this differs conceptually from our dynamic post-CPB hemodynamic indices, it illustrates the broader interest in complementary perioperative risk markers. Taken together, our findings suggest that post-CPB SI and MSI may provide prognostic information beyond baseline surgical risk, but their incremental value for mortality discrimination remains uncertain and requires external validation.

Beyond mortality, age-adjusted shock indices were associated with selected postoperative complications in our cohort. Higher post-CPB Age-SI and Age-MSI were associated particularly with surgical re-exploration and overall postoperative morbidity, although their discriminatory performance was modest. Re-exploration after cardiac surgery is itself associated with clinically important adverse outcomes, emphasizing the relevance of identifying perioperative markers associated with this complication [13]. Because the original OPM composite included postoperative hypertension and hypotension, which overlap conceptually with the blood-pressure component of the shock indices, we repeated the analysis after excluding these components.

The associations persisted after exclusion of postoperative hypertension and hypotension; however, discrimination remained modest, indicating that statistical association should not be interpreted as evidence of clinically meaningful predictive performance for the modified composite outcome.

Postoperative AF is multifactorial and has also been studied using inflammatory markers; Demir reported an association between the systemic inflammatory response index and postoperative AF in CABG patients [14]. In our cohort, Age-SI and Age-MSI showed only modest univariable discrimination for AF, and the associations were attenuated after adjustment for EuroSCORE II. These findings should not be interpreted as evidence of a common mechanism. SI and MSI were also associated with reintubation, but the small number of events precluded multivariable analysis. Associations with mechanical ventilation duration were weak, and no significant correlations were observed with ICU length of stay.

Post-CPB hemodynamics should also be interpreted in the context of the institutional perfusion strategy. All patients in the present study underwent conventional extracorporeal circulation because MiECC was not available at our institution during the study period. Studies comparing conventional extracorporeal circulation with MiECC indicate that perfusion strategy can influence immediate postoperative physiology and clinical outcomes [15]. In addition, recent isolated-CABG data using MiECC have identified perioperative factors associated with prolonged ICU stay [16]. These studies were conducted in different perfusion settings and do not directly validate our findings; rather, they underscore that post-CPB shock indices should be interpreted within the perfusion context in which they were measured.

Postoperative AKI was another outcome for which an association with an age-adjusted shock index was observed, although the discriminatory performance of Age-SI was modest. Cardiac surgery-associated AKI is multifactorial, and perioperative hemodynamic disturbances and impaired renal perfusion are among the factors implicated in its development [17]. Consistent with this concept, Srivastava et al. recently reported that greater postoperative deficits in renal perfusion pressure and mean arterial pressure were associated with an increased risk of AKI after cardiac surgery with CPB [18]. Although Age-SI does not directly measure renal perfusion, its association with AKI in our cohort may therefore reflect, at least in part, the broader hemodynamic burden during the early post-CPB period. This finding remains exploratory and should not be interpreted as evidence that Age-SI is a specific marker of renal injury. Moreover, our prespecified AKI classification did not incorporate the KDIGO criterion of an absolute serum creatinine increase of ≥0.3 mg/dL within 48 h, which may have resulted in under-recognition of milder stage 1 AKI. The observed association should therefore be interpreted cautiously and requires confirmation in studies specifically designed to evaluate postoperative AKI.

From a clinical perspective, SI and MSI are simple indices derived from routinely monitored hemodynamic variables and can be calculated without additional equipment or laboratory testing. Their assessment after separation from CPB may provide a practical signal of hemodynamic instability at a clinically important transition point. However, their limited incremental discrimination beyond EuroSCORE II and the low positive predictive values of the internally derived thresholds argue against their use as standalone decision tools. Rather, these indices may be most useful as adjunctive markers that prompt closer clinical assessment in conjunction with established risk factors and the overall hemodynamic context. The Youden-derived cut-offs should be regarded as exploratory and should not be used as treatment thresholds without external validation.

This study has several limitations. First, its retrospective, single-center design limits generalizability and precludes causal inference. Although adjustment for EuroSCORE II was performed, residual confounding from ventricular function, systemic inflammatory state, procedural complexity, and other measured or unmeasured factors cannot be excluded. In particular, LVEF was lower among non-survivors, but the limited number of deaths precluded inclusion of additional covariates without substantial overfitting. Second, only 12 in-hospital deaths occurred overall and 10 were available for post-CPB analyses. Mortality models were therefore deliberately parsimonious. Although Firth penalized regression and bootstrap internal validation supported the robustness of the observed associations, these methods cannot overcome the limited event count, and the incremental predictive performance beyond EuroSCORE II remains uncertain. Third, two patients who could not be separated from CPB had no post-CPB measurement, and later measurements were structurally unavailable after death. This outcome-related missingness may introduce selection bias; therefore, post-CPB findings apply only to patients who reached that assessment point. Fourth, hemodynamic measurements were influenced by perioperative management, including fluid administration and vasoactive or inotropic therapy, for which detailed dose and time-resolved exposure data were unavailable. Fifth, the prespecified AKI classification did not incorporate the absolute ≥0.3 mg/dL serum creatinine increase within 48 h included in the full KDIGO definition and may therefore have under-recognized mild AKI. Sixth, the OPM composite included heterogeneous complications and remains exploratory despite the sensitivity analysis excluding postoperative hypertension and hypotension. Finally, multiple indices, time points, and secondary outcomes were evaluated without formal adjustment for multiple comparisons, and the internally derived mortality cut-offs have not been externally validated. Accordingly, secondary associations and proposed thresholds should be considered hypothesis-generating rather than confirmatory.

## 5. Conclusions

In patients undergoing elective isolated CABG, post-CPB SI and MSI were associated with in-hospital mortality among those who reached the post-CPB assessment point and remained associated after adjustment for EuroSCORE II. Although addition of either index improved model fit, the incremental improvement in discrimination was modest and uncertain. Age-adjusted indices also showed exploratory associations with selected postoperative morbidity outcomes. These readily available indices may serve as supportive markers of perioperative hemodynamic instability rather than standalone prognostic tools. Given the limited number of mortality events, outcome-related missingness, and lack of external validation, these findings should be considered hypothesis-generating. Larger prospective multicenter studies are needed to determine whether post-CPB shock indices provide clinically meaningful prognostic information beyond established cardiac surgical risk models and whether their incorporation into perioperative assessment improves clinical decision-making.

## Figures and Tables

**Table 1:** Baseline and intraoperative characteristics according to in-hospital mortality.

Characteristic	Survivors (n = 233)	Non-Survivors (n = 12)	*p*-Value
Age, years	60 [54–67]	65.5 [60.5–71.3]	0.098
Sex			0.469
Female	50 (21.5)	1 (8.3)	
Male	183 (78.5)	11 (91.7)	
BMI, kg/m^2^	27.34 [25.10–29.75]	26.44 [25.79–27.49]	0.286
BSA, m^2^	1.87 [1.77–1.97]	1.81 [1.70–1.84]	**0.043**
NYHA functional class			**<0.001**
Class I	28 (12.0)	0 (0)	
Class II	145 (62.2)	3 (25.0)	
Class III	58 (24.9)	8 (66.7)	
Class IV	2 (0.9)	1 (8.3)	
LVEF, %	55 [50–60]	50 [41.3–55]	**0.023**
EuroSCORE II, %	1.08 [0.80–1.59]	2.93 [1.61–6.56]	**<0.001**
Diabetes mellitus	115 (49.4)	5 (41.7)	0.769
Hypertension	148 (63.5)	11 (91.7)	0.061
Preoperative renal failure	7 (3.0)	3 (25.0)	**0.009**
Previous cerebrovascular event	6 (2.6)	1 (8.3)	0.300
Pulmonary arterial hypertension	2 (0.9)	0 (0)	1.000
Preoperative atrial fibrillation	1 (0.4)	0 (0)	1.000
Smoking	124 (53.2)	4 (33.3)	0.239
COPD	31 (13.3)	1 (8.3)	1.000
Preoperative hemoglobin, g/dL	14.0 [13.0–15.0]	13.8 [11.8–14.3]	0.373
Preoperative platelet count, ×10^3^/µL	251 [212–296]	202.5 [185.3–258.5]	0.072
Preoperative INR	1.03 [0.98–1.09]	1.09 [1.01–1.15]	0.092
Preoperative urea, mg/dL	38 [31–45]	53 [40–76]	**0.007**
Preoperative creatinine, mg/dL	0.82 [0.70–1.00]	1.03 [0.84–2.30]	**0.011**
Cross-clamp time, min	61 [44–77]	72 [45.3–94]	0.169
CPB duration, min	94 [75–115]	114 [81.8–139]	0.096
Anesthesia duration, min	240 [210–300]	230 [200–300]	0.858
Surgery duration, min	210 [180–255]	197.5 [180–272.5]	0.766
Intraoperative urine output, mL	850 [550–1250]	750 [102.5–1225]	0.370
Intraoperative peak lactate, mmol/L	2.84 [2.28–3.60]	2.86 [2.62–5.01]	0.223
Any intraoperative inotrope/vasopressor	184 (79.0)	12 (100)	0.132
Intraoperative norepinephrine	140 (60.1)	12 (100)	**0.004**
Intraoperative dopamine	165 (70.8)	10 (83.3)	0.517

Data are presented as median [interquartile range] or n (%), as appropriate. Continuous variables were compared using the Mann–Whitney U test. Binary categorical variables were compared using Fisher’s exact test when appropriate because of the small number of deaths; NYHA functional class was compared across categories. BMI: body mass index; BSA: body surface area; NYHA: New York Heart Association; LVEF: left ventricular ejection fraction; COPD: chronic obstructive pulmonary disease; INR: international normalized ratio; CPB: cardiopulmonary bypass. Bold values indicate statistical significance (*p* < 0.05).

**Table 2:** Discrimination of post-CPB shock indices and EuroSCORE II for in-hospital mortality.

Predictor	Analysis Set, n/Deaths	AUC (95% CI)	Exploratory Youden Cut-Off *	Sensitivity	Specificity	PPV	NPV
Post-CPB SI	243/10	0.798 (0.621–0.974)	≥1.31	70.0%	88.4%	20.6%	98.6%
Post-CPB MSI	243/10	0.793 (0.606–0.980)	≥1.87	70.0%	85.4%	17.1%	98.5%
Post-CPB Age-SI	243/10	0.742 (0.554–0.929)	≥76.87	70.0%	79.0%	12.5%	98.4%
Post-CPB Age-MSI	243/10	0.733 (0.527–0.939)	≥126.69	60.0%	90.1%	20.7%	98.1%
EuroSCORE II	245/12	0.830 (0.698–0.963)	≥1.47	91.7%	69.1%	13.3%	99.4%

AUC: area under the receiver operating characteristic curve; CI: confidence interval; SI: shock index; MSI: modified shock index; Age-SI: age shock index; Age-MSI: age-modified shock index; CPB: cardiopulmonary bypass; EuroSCORE II: European System for Cardiac Operative Risk Evaluation II; PPV: positive predictive value; NPV: negative predictive value. * Cut-off values were determined using the Youden index and are exploratory, internally derived thresholds rather than externally validated clinical cut-offs. Post-CPB shock-index analyses included 243 patients with 10 deaths because two non-survivors could not be successfully separated from CPB and therefore had no post-CPB measurement. EuroSCORE II values in this table refer to the entire cohort (n = 245; 12 deaths). Direct comparisons with post-CPB indices and incremental models were performed separately in the common complete-case–cohort (n = 243; 10 deaths).

**Table 3:** Associations between post-CPB shock indices and postoperative outcomes.

Outcome	Events, n/N (%) or Value	Main Post-CPB Finding	AUC or Correlation Coefficient	Adjusted Association *
Acute kidney injury	28/244 (11.5)	Age-SI: 75.32 vs. 58.45 (AKI vs. no AKI); *p* = 0.023	Age-SI: 0.635	—
Postoperative AF	36/245 (14.7)	Age-SI: *p* = 0.037; Age-MSI: *p* = 0.028	Age-SI: 0.623; Age-MSI: 0.629	Age-SI: aOR 1.15/10 units, *p* = 0.089; Age-MSI: aOR 1.11/10 units, *p* = 0.070
Postoperative stroke	2/244 (0.8)	Inferential analysis not performed †	—	—
Re-exploration	25/245 (10.2)	Age-SI and Age-MSI: both *p* < 0.001	Age-SI: 0.710; Age-MSI: 0.720	Age-SI: aOR 1.29 (95% CI 1.08–1.53), *p* = 0.005; Age-MSI: aOR 1.22 (95% CI 1.07–1.39), *p* = 0.002
Reintubation	10/243 (4.1)	SI: *p* = 0.0048; MSI: *p* = 0.0034	SI: 0.764; MSI: 0.774	Not performed ‡
Original OPM	155/245 (63.3)	Age-SI and Age-MSI: both *p* = 0.005	Age-SI: 0.607; Age-MSI: 0.607	Age-SI: aOR 1.23 (95% CI 1.05–1.43), *p* = 0.008; Age-MSI: aOR 1.17 (95% CI 1.05–1.30), *p* = 0.005
Modified OPM §	120/245 (49.0)	Age-SI: 64.44 [51.99–80.55] vs. 55.70 [48.35–67.23], *p* < 0.001; Age-MSI: 93.53 [74.71–116.43] vs. 82.31 [68.54–96.59], *p* < 0.001	Age-SI: 0.627; Age-MSI: 0.625	Age-SI: aOR 1.25 (95% CI 1.08–1.44), *p* = 0.002; Age-MSI: aOR 1.17 (95% CI 1.06–1.29), *p* = 0.002
ICU length of stay, days	2 [2, 3]	No significant association	ρ = 0.103–0.112; all *p* > 0.05	—
Mechanical ventilation duration, h	5.92 [4.50–7.00]	Age-SI and Age-MSI: weak positive correlations; both *p* = 0.024	Age-SI: ρ = 0.146; Age-MSI: ρ = 0.146	—

AUC: area under the receiver operating characteristic curve; SI: shock index; MSI: modified shock index; Age-SI: age shock index; Age-MSI: age-modified shock index; CPB: cardiopulmonary bypass; AKI: acute kidney injury; AF: atrial fibrillation; OPM: overall postoperative morbidity; ICU: intensive care unit; aOR: adjusted odds ratio; CI: confidence interval; ρ: Spearman’s rank correlation coefficient. * Adjusted models included EuroSCORE II and the indicated post-CPB shock index only. Adjusted odds ratios for Age-SI and Age-MSI are expressed per 10-unit increase. Secondary outcome analyses were exploratory and were not adjusted for multiple comparisons. † Postoperative stroke occurred in two patients; only one had an available post-CPB shock-index measurement, precluding meaningful inferential analysis. One AKI assessment was unavailable because of early death before postoperative AKI could be evaluated. ‡ Multivariable analysis was not performed for reintubation because of the small number of events. § Modified OPM excluded postoperative hypertension and hypotension from the original composite definition to reduce predictor–outcome overlap. Modified OPM occurred in 120/245 patients overall; 119/243 patients with available post-CPB measurements met the modified definition.

## Data Availability

The datasets generated and/or analyzed during the current study are not publicly available due to patient confidentiality and ethical restrictions. De-identified data may be made available from the corresponding author upon reasonable request, subject to institutional and ethical approval.

## Supplementary Materials

The following supporting information can be downloaded at: https://www.mdpi.com/article/10.3390/jcm15218629/s1, Table S1. Perioperative shock index parameters according to in-hospital mortality.
