# Supplementary material for: Association of Perioperative Shock Indices with In-Hospital Mortality and Postoperative Morbidity After Coronary Artery Bypass Grafting: A Retrospective Cohort Study
Source: J Clin Med. 2026 Nov 5;15(21):8629. doi: 10.3390/jcm15218629 (PMC13764082; doi:10.3390/jcm15218629)
Supplement: Supplementary file 1 [file jcm-15-08629-s001.zip › jcm-4647138-supplementary.pdf]

Supplementary Table S1. Perioperative shock index parameters according to in-hospital mortality

Time point / Index	Survivors	Non-survivors	p value
Preoperative			
SI	0.536 [0.469–0.620] (n=233)	0.559 [0.471–0.745] (n=10)	0.510
MSI	0.750 [0.674–0.876] (n=233)	0.807 [0.629–0.950] (n=11)	0.932
Age-SI	31.80 [27.33–37.57] (n=233)	32.21 [30.55–43.97] (n=10)	0.364
Age-MSI	45.00 [39.42–52.83] (n=233)	41.38 [39.81–60.41] (n=11)	0.770
Pre-CPB			
SI	0.672 [0.571–0.851] (n=233)	0.745 [0.647–0.864] (n=10)	0.208
MSI	1.000 [0.841–1.184] (n=233)	1.117 [0.939–1.179] (n=10)	0.154
Age-SI	40.84 [33.66–50.93] (n=233)	46.63 [38.45–58.89] (n=10)	0.193
Age-MSI	60.44 [47.44–71.21] (n=233)	68.82 [57.67–85.10] (n=10)	0.147
Post-CPB			
SI	1.023 [0.849–1.165] (n=233)	1.343 [1.201–1.538] (n=10)	0.001
MSI	1.477 [1.256–1.700] (n=233)	2.030 [1.726–2.073] (n=10)	0.002
Age-SI	58.76 [49.50–72.73] (n=233)	83.24 [62.34–110.08] (n=10)	0.010
Age-MSI	87.54 [69.69–106.45] (n=233)	127.29 [88.57–147.35] (n=10)	0.013
Postoperative 0 h			
SI	0.879 [0.728–1.084] (n=232)	0.960 [0.712–1.175] (n=10)	0.533
MSI	1.310 [1.109–1.588] (n=232)	1.395 [1.069–1.707] (n=10)	0.636
Age-SI	52.57 [42.46–64.30] (n=232)	57.09 [44.79–63.33] (n=10)	0.397
Age-MSI	77.88 [63.62–95.45] (n=232)	81.86 [71.54–95.82] (n=10)	0.541
Postoperative 6 h			
SI	0.833 [0.750–0.926] (n=232)	0.840 [0.772–0.932] (n=6)	0.900
MSI	1.235 [1.106–1.375] (n=231)	1.235 [1.235–1.320] (n=5)	0.987
Age-SI	50.00 [43.29–57.97] (n=232)	54.59 [49.95–58.16] (n=6)	0.451
Age-MSI	73.41 [63.75–84.51] (n=231)	77.82 [72.00–86.47] (n=5)	0.776
Postoperative 12 h			
SI	0.818 [0.737–0.913] (n=232)	0.813 [0.800–0.859] (n=6)	0.912
MSI	1.179 [1.071–1.360] (n=232)	1.200 [1.086–1.359] (n=6)	0.769
Age-SI	48.92 [43.20–56.42] (n=232)	56.06 [45.76–59.40] (n=6)	0.524
Age-MSI	70.49 [62.41–84.18] (n=232)	77.19 [64.67–94.90] (n=6)	0.517
Postoperative 24 h			
SI	0.781 [0.695–0.857] (n=232)	0.762 [0.707–0.821] (n=6)	0.732
MSI	1.151 [1.022–1.251] (n=232)	1.019 [0.991–1.130] (n=6)	0.273
Age-SI	46.81 [40.00–53.32] (n=232)	49.83 [44.87–51.80] (n=6)	0.670
Age-MSI	69.04 [59.65–78.15] (n=232)	65.63 [64.46–70.01] (n=6)	0.817
